# Temperature dependence of dielectric properties of blood at 10 Hz–100 MHz

**DOI:** 10.3389/fphys.2022.1053233

**Published:** 2022-10-26

**Authors:** Weice Wang, Weichen Li, Benyuan Liu, Lei Wang, Kun Li, Yu Wang, Zhenyu Ji, Canhua Xu, Xuetao Shi

**Affiliations:** ^1^ Shaanxi Provincial Key Laboratory of Bioelectromagnetic Detection and Intelligent Perception, Department of Biomedical Engineering, Air Force Medical University, Xi’an, China; ^2^ School of Life Sciences, Northwest University, Xi’an, China; ^3^ Institute of Medical Research, Northwestern Polytechnical University, Xi’an, China; ^4^ Faculty of Electrical and Control Engineering, Liaoning Technical University, Huludao, China

**Keywords:** blood, dielectric properties, bioelectromagnetism, bioimpedance, temperature dependence

## Abstract

The temperature dependence of the dielectric properties of blood is important for studying the biological effects of electromagnetic fields, electromagnetic protection, disease diagnosis, and treatment. However, owing to the limitations of measurement methods, there are still some uncertainties regarding the temperature characteristics of the dielectric properties of blood at low and medium frequencies. In this study, we designed a composite impedance measurement box with high heat transfer efficiency that allowed for a four/two-electrode measurement method. Four-electrode measurements were carried out at 10 Hz-1 MHz to overcome the influence of electrode polarization, and two-electrode measurements were carried out at 100 Hz-100 MHz to avoid the influence of distribution parameters, and the data was integrated to achieve dielectric measurements at 10 Hz-100 MHz. At the same time, the temperature of fresh blood from rabbits was controlled at 17–39°C in combination with a temperature-controlled water sink. The results showed that the temperature coefficient for the real part of the resistivity of blood remained constant from 10 Hz to 100 kHz (−2.42%/°C) and then gradually decreased to −0.26%/°C. The temperature coefficient of the imaginary part was positive and bimodal from 6.31 kHz to 100 MHz, with peaks of 5.22%/°C and 4.14%/°C at 126 kHz and 39.8 MHz, respectively. Finally, a third-order function model was developed to describe the dielectric spectra at these temperatures, in which the resistivity parameter in each dispersion zone decreased linearly with temperature and each characteristic frequency increased linearly with temperature. The model could estimate the dielectric properties at any frequency and temperature in this range, and the maximum error was less than 1.39%, thus laying the foundation for subsequent studies.

## Introduction

The dielectric properties of biological tissues are characterized by their absorption and coupling of electromagnetic energy in electromagnetic fields. These are the fundamental physical properties of tissues that contain much physiological and pathological information and are significantly influenced by frequency, temperature, and other factors. Blood is an important biological tissue throughout the body, and the variation in its dielectric properties with frequency can be used to monitor abnormal physiological changes occurring in the body (Abdalla, 2011; Basey-Fisher et al., 2014). It can also be used in medical applications, such as specific absorption rate calculations, dose assessments, and other therapeutic and diagnostic applications of electromagnetic fields (Salahuddin et al., 2017b) (e.g., electrical impedance tomography (EIT), magnetic induction tomography (MIT), and microwave imaging (MI)). Therefore, precise knowledge of the dielectric properties of blood and their changing rules under specific conditions can help us understand the biological effects of electromagnetic fields and provide the basic spectral data required for electromagnetic imaging.

In applications, such as radiofrequency thermal ablation (Deas Yero et al., 2018), microwave thermal ablation (Ley et al., 2019; Sebek et al., 2019; Zia et al., 2021), cryoablation (Fischer et al., 2019), and non-invasive temperature monitoring techniques based on tissue dielectric properties (Ley et al., 2019), it is essential to precisely master the dielectric properties of each tissue or organ and their variations with temperature. Blood perfusion is the main cause of temperature variations in each tissue (Rossmann and Haemmerich, 2014). Therefore, it is necessary to first investigate the temperature dependence of the dielectric properties of blood. It is important to accurately understand the temperature dependence of the dielectric properties of blood to help us understand the biophysical laws by which temperature affects tissues and accurately model the temperature characteristics of the dielectric parameters of organs in vivo. It also laid the foundation for diagnosing and treating diseases, calculating the specific absorption rate of the body (Deas Yero et al., 2018), and studying the bioeffects of electromagnetic fields, electromagnetic protection, and early warning of brain damage based on dielectric parameters (Li et al., 2018; Liu et al., 2019) in our group’s work on cardiac surgery (Rajaram et al., 2020) when temperature changes (Englum et al., 2017).

Many studies have been carried out on the dielectric properties of blood. For example, Abdalla(Abdalla, 2011) studied the dielectric properties of blood at different glucose concentrations for the rapid diagnosis of diabetes, and Basey-Fisher et al. (Basey-Fisher et al., 2014) investigated the relationship between blood dielectric properties and hemoglobin concentration at 200 MHz-40 GHz as a means to achieve independent and minimally invasive monitoring of hemoglobin concentration for rapid diagnosis of diseases such as anemia. In order to provide basic spectral data for electromagnetic medicine applications, Salahuddin et al. (Salahuddin et al., 2017a; Salahuddin et al., 2017b) investigated the dielectric properties of fresh blood at 400 MHz-20 GHz. However, relatively few studies have been conducted on their temperature dependence. For example, to calculate the cardiac output in patients using the electrical impedance method, Mohapatra and Hill (Mohapatra and Hill, 1975) investigated the variation of blood resistivity with temperature (22–40°C) at 100 kHz using an electrical bridge method, and they established a relationship between resistivity and temperature; for the purpose of microwave non-invasive temperature monitoring, Ley et al.(2019) investigated the variation of blood dielectric parameters with temperature (30–50°C) at 0.5–7 GHz and a double-order Cole-Cole model was established; to calculate the specific absorption rate, the temperature (25–45°C) sensitivity of the blood dielectric parameters was investigated at 1 MHz–1 GHz using the terminal coaxial reflection method (Jaspard and Nadi, 2002); while Wolf et al.(2011) investigated the temperature dependence (6.85–56.85°C) of blood dielectric parameters at 1 Hz–40 GHz using the two-electrode method and the coaxial reflection method, however, the authors did not address the severe effect of electrode polarisation at low frequencies. In summary, few studies have been conducted on the temperature dependence of low-frequency dielectric parameters of blood, and low-frequency data are severely affected by electrode polarization owing to the limitations of the measurement method. In addition, few relevant studies have focused on the effect of time away from the body on the blood dielectric parameters. Simultaneously, the dielectric properties at 10 Hz–100 MHz can reflect the internal characteristics of tissues, cells, and macromolecules. Both physiological and pathological changes are reflected in these parameters in this frequency range (Ackman et al., 1976). The study of dielectric parameters in the 10 Hz–100 MHz range is of great theoretical and practical value, and many applications, such as EIT, MIT, and radiofrequency ablation, operate in this frequency range. Our research was designed to address these issues.

Therefore, in the context of the apparent impact of blood temperature changes on dielectric parameters during cardiac surgery (Li et al., 2018; Liu et al., 2019), we established a platform for measuring the dielectric properties of active biological tissues. The platform is suitable for investigating the temperature dependence of dielectric properties and overcomes the effects of low-frequency electrode polarization and high-frequency interlead distribution parameters. Firstly, the dielectric properties of physiological saline at the corresponding temperature at 10 Hz-100 MHz were measured to verify the temperature control effect and measurement accuracy of the platform. Secondly, the time dependence of blood dielectric properties and the influence of cooling and rewarming process on blood dielectric parameters were studied. On the basis of mastering the influence of *in vitro* time and cooling and rewarming process on blood dielectric parameters, we conducted a temperature (17–39°C) study of the dielectric properties of blood at 10 Hz–100 MHz using this platform. Further, We extracted the temperature coefficient of the dielectric parameters of the blood. A third-order function model consisting of the relationship between the dielectric parameters, frequencies, and temperatures was established, laying the foundation for investigating the biophysical laws of temperature affecting the dielectric parameters of tissues.

## Materials and methods

### Construction of a platform

For the important 10 Hz–100 MHz, conventional methods are not adequate for the high-accuracy measurement of dielectric parameters in this frequency range. This is due to factors, such as electrode polarization, contact impedance at low frequencies, and distributed parameters, such as stray capacitances between wires in the higher frequency range. Therefore, we developed a combined four/two-electrode measurement method (Wang et al., 2022).

**FIGURE 1:**
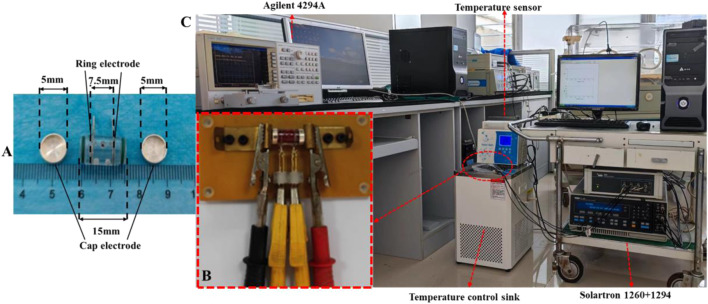
**(A)** Impedance measurement box for dielectric parameter measurement. **(B)** Fixture for four-electrode measurement. **(C)** Platform suitable for the investigation of the temperature dependence of dielectric properties of active biological tissues.

The method is based on a composite impedance measurement box that has high heat transfer efficiency and can measure impedance using a four/two-electrode method (Figure 1A). A four-electrode fixture (Figure 1B) was designed for connection to the four-electrode measuring instrument. At low frequencies, a Solartron 1260 electrical impedance analyzer + Solartron 1294A bioimpedance measurement interface (Schlumberger, United Kingdom) was used with a four-electrode fixture to perform the four-electrode measurements at 10 Hz-1 MHz, where the silver/silver chloride cap was used as the excitation electrode and the silver ring in the middle was used as the measurement electrode. At high frequencies, two-electrode measurements were made at 100 Hz–100 MHz using the Agilent 4294A+ Agilent 16092A fixture (Agilent Technologies, United States), where the silver/silver chloride cap was used as the excitation electrode as well as the measurement electrode.

The four-electrode fixture connected to the Solartron 1260 + 1294A impedance analyzer was sealed with a polyethylene film and immersed in a temperature-controlled water sink (Shanghai Beiyi Testing Instruments Company, DC-0506, accuracy ±0.1°C) for temperature control (Figure 1C). The target temperatures were set at 17, 22, 27, 32, 37, and 39°C. The temperature was monitored using a miniature temperature probe (JRD-C-I, Jiangsu Jireida Electronics Company) inserted into the hole outside the impedance measurement box. Four-electrode measurements were performed when the target temperature was reached. After completion, the box was quickly transferred to an Agilent 16092A fixture, which was covered with the same temperature insulation bag for the two-electrode measurements (Wang et al., 2022).

### Sample collection and experimental methods

The experimental subjects were healthy adult New Zealand rabbits weighing (2.6 ± 0.3) kg, and males and females were randomly assigned to the following groups. Group A: Blood samples from six rabbits were used to study the changes in their dielectric parameters over time; Group B: Blood samples from six rabbits were used to study the effects of cooling and rewarming processes on the dielectric properties of blood; Group C: Blood samples from eight rabbits were used to study the temperature dependence of blood dielectric parameters. Before the experiment, rabbits were deprived of food for 4 h and water for 2 h. Anesthesia was induced with isoflurane using a respiratory anesthesia machine. Blood samples were collected from a vein at the edge of the ear and temporarily preserved in vacuum anticoagulation tubes with sodium heparin. Blood was rapidly transferred to an impedance measurement box for temperature control and subsequent dielectric measurements after 3 min of isolation.

### Data analysis

The raw data were the real (*Re*) and imaginary (*Im*) parts of the impedance. The real (*ρ*_*re*_) and imaginary (*ρ*_*im*_) parts of the resistivity of the sample can be derived from the geometric parameters of the measurement box (as Eq. 1, 2), where *S* is the cross-sectional area of the sample under test and *l* is the effective length of the sample under test (the distance between the two silver/silver chloride cap electrodes for the two-electrode method or the distance between the middle two silver ring electrodes for the four-electrode method).

ρre=Re⋅Sl⋅100 (1)

ρim=Im⋅Sl⋅100 (2)

To investigate the temperature sensitivity of the dielectric parameters, the measurements at 17, 22, 27, 32, 37, and 39°C were fitted with the least-squares method to establish the dielectric properties as a function of temperature.

### Mathematical model of the temperature-frequency dependent blood dielectric properties

**FIGURE 2:**
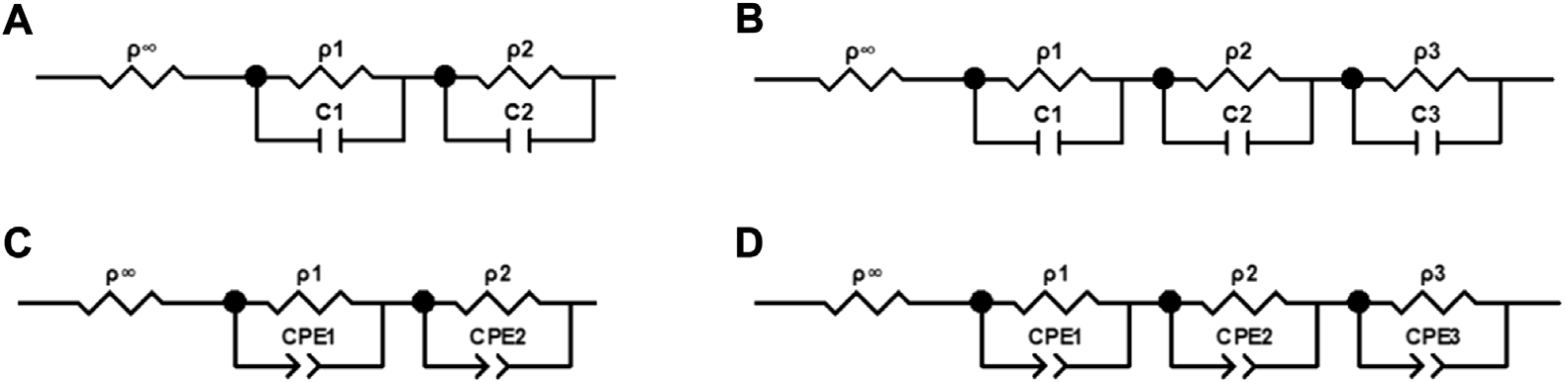
Equivalent circuits of complex electrical resistivity of blood. **(A)** Circuit containing two parallel resistors and capacitors. **(B)** Circuit containing three parallel resistors and capacitors. **(C)** Circuit containing two parallel resistors and constant phase elements. **(D)** Circuit containing three parallel resistors and constant phase elements.

To further investigate the temperature and frequency dependence of the blood dielectric parameters, we built equivalent circuits with parallel resistors and capacitors based on the multi-order “three-element” equivalent circuit model (Burtscher et al., 2020) or parallel resistors and constant phase angle cells based on the multi-order Cole-Cole equivalent model, as shown in Figure 2. We estimated the model parameters of the blood complex resistivities using the *Zview* software (*Zview*, 2.70) and assessed the relative error between the absolute values of the complex resistivity calculated by the equivalent circuit model and the measured values using Eq. 3.

$$Err=\frac{\left|\rho_{measured}\right|-\left|\rho_{fit}\right|}{\left|\rho_{measured}\right|}*100\%$$ (3)

Finally, the best model was selected based on Eq. 3, and the temperature dependence of each characteristic parameter in this model was investigated to establish a mathematical model that can characterize the temperature and frequency dependence of the dielectric properties of blood.

## Results

### Validation of the measurement accuracy of the broadband dielectric measurement platform

To verify the measurement accuracy and temperature control effect of the dielectric measurement platform (Wang et al., 2022), Supplementary Figure 1 showed the variation of the mean conductivities of six physiological saline samples at different temperatures from 39°C to 17°C with frequencies (the error bars are standard deviations), and each test took 35.3 ± 3.1 min. As we can see, the saline conductivities did not change significantly with frequency and were significantly influenced by temperature. Supplementary Table S1 gave a comparison between the theoretical values of the physiological saline conductivities at 32, 27, 22, and 17°C (Peyman et al., 2007) and the average values of conductivity from this measurement over the entire frequency range at the corresponding temperature, which showed a maximum error of only 1.73%.

**FIGURE 3:**
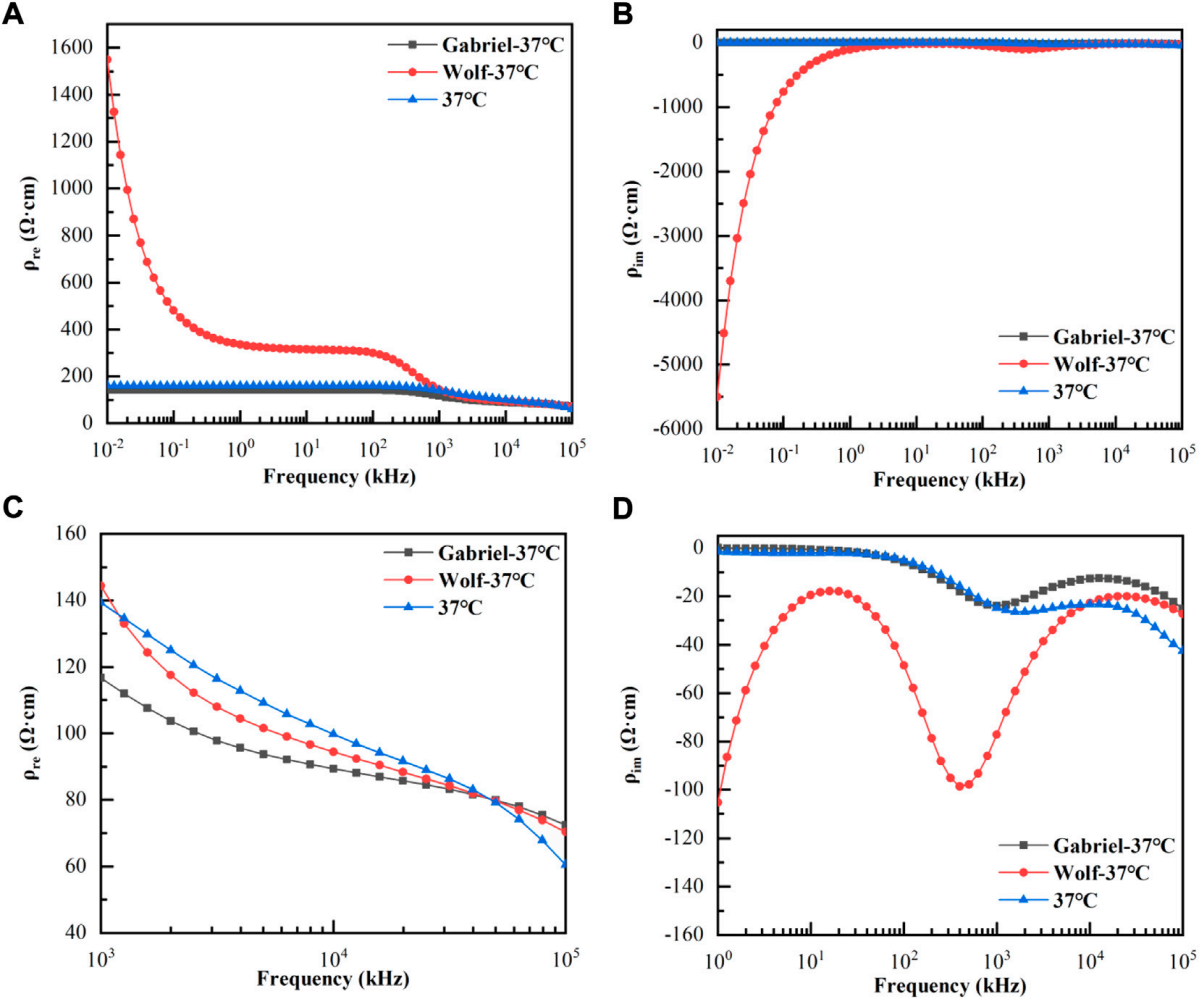
Variation of the real and imaginary parts of the blood resistivities with frequencies at 37°C. **(A)**: Real parts of resistivities at 10 Hz–100 MHz; **(B)**: Imaginary parts of resistivities at 10 Hz–100 MHz; **(C)**: Real parts of resistivities at 1 kHz–100 MHz; **(D)**: Imaginary parts of resistivities at 1 kHz–100 MHz.

Figures 3A,B were shown as the variation of the real and imaginary parts of the blood resistivities of (Gabriel et al., 1996), (Wolf et al., 2011), and our measurement at 37°C with 10–100 MHz. Figure 3C displayed a magnified view of the change in the real part of the resistivity above 1 MHz, and Figure 3D gave a magnified view of the change in the imaginary part above 1 kHz. As shown, the real part of the resistivity measured by Wolf at < 1 MHz was much larger than that measured by Gabriel and us, whereas the three values at > 1 MHz were more or less the same. In addition, the values measured by Gabriel and us were close to each other throughout the frequency range, with the same trend. The imaginary part measured by Wolf at < 10 kHz was also much larger than that measured by Gabriel and us; the latter two converged to zero at < 10 kHz, and the trend was more consistent throughout the frequency range.

### Time dependence of the dielectric properties of the isolated blood

To prevent denaturation from affecting the dielectric parameters when the blood was left for too long, we first investigated the variation in the blood dielectric parameters with time. Supplementary Figure 2 showed that the mean resistivity of the real parts of the six blood samples changed with the time of separation at 37°C at 10 Hz, 1 kHz, 50 kHz, 500 kHz, 1 MHz, 10 MHz, and 100 MHz. The real part gradually increased with time. We used the real part of the resistivity at 10 min of separation as the reference point and calculated the relative rate of change at each time point; the maximum relative rate of change at each frequency point at 20, 30, 40, 60, 80, 100, 120, 140, 160, 180, 200, and 220 min of separation were 0.69%, 0.64%, 0.97%, 1.13%, 1.22%, 1.85%, 2.45%, 2.87%, 3.31%, 4.23%, 5.12%, and 5.14%, respectively.

### Effect of cooling and rewarming processes on the dielectric properties of blood

**FIGURE 4:**
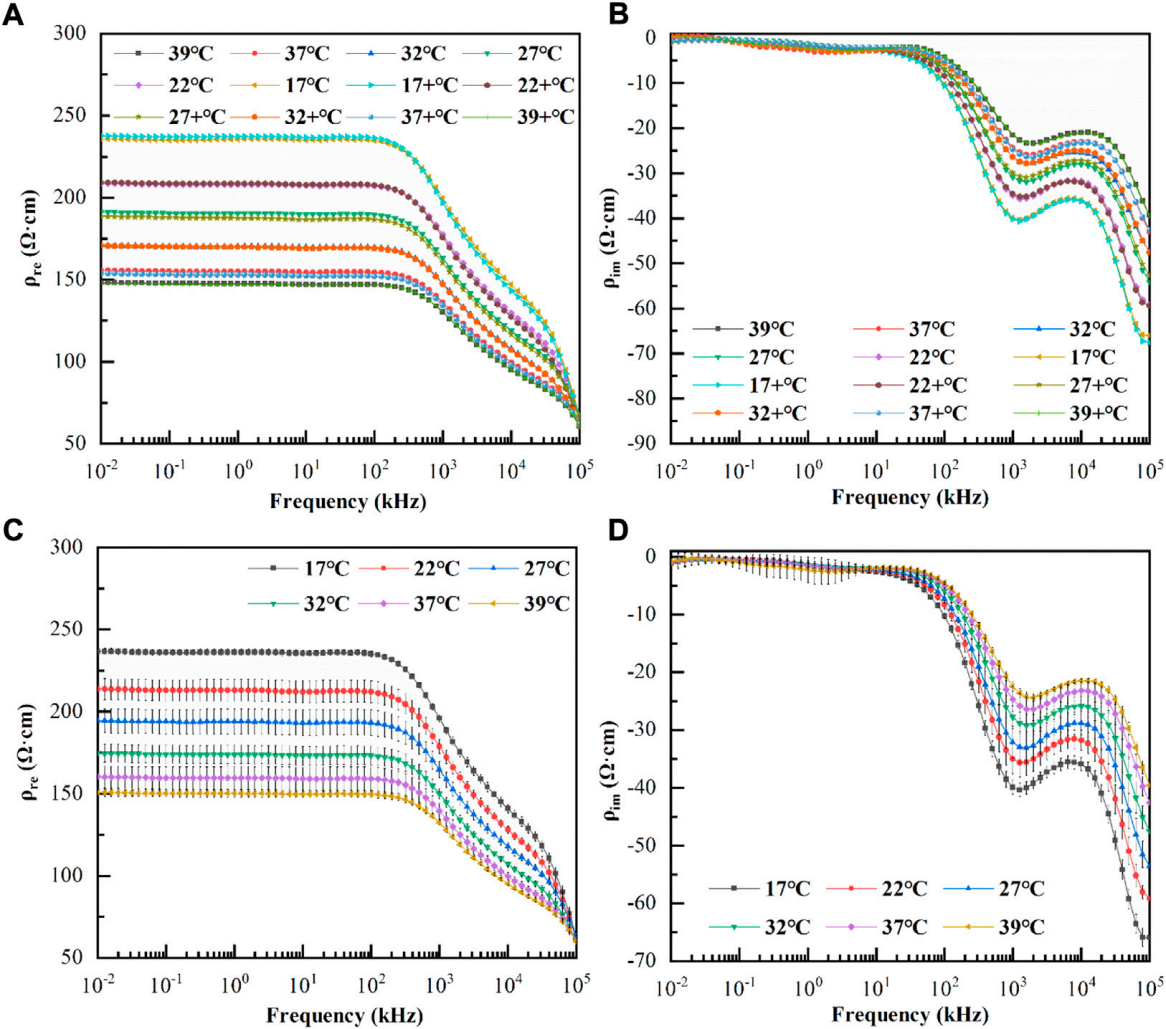
**(A)**: Variation of the real parts of resistivity with frequency during cooling and rewarming; **(B)**: Variation of the imaginary parts of resistivity with frequency during cooling and rewarming; 39°C, 37°C, 32°C, 27°C, 22°C, and 17 °C indicate the corresponding temperatures during the cooling process, while 17+ °C, 22+ °C, 27+ °C, 32+ °C, 37+ °C, and 39+ °C indicate the corresponding temperatures during the rewarming process. **(C)**: Variation of the mean value of the real parts of resistivity with frequency at each temperature (The error bars are standard deviations); **(D)**: Variation of the mean value of the imaginary parts of resistivity with frequency at each temperature (The error bars are standard deviations).

Most cardiac surgeries involve both cooling and rewarming procedures, and whether the temperature characteristics of blood dielectric parameters are consistent during cooling and rewarming is rarely observed. Therefore, we investigated the variations in the dielectric parameters of six blood samples with frequency during cooling and rewarming. Figure 4A,B displayed the variations in the mean values of the real and imaginary parts of the resistivity at each temperature with frequency during the first cooling and then rewarming, respectively. Here, 39, 37, 32, 27, 22, and 17°C indicate the corresponding temperatures during the cooling process, while 17+, 22+, 27+, 32+, 37+, and 39+°C indicate the corresponding temperatures during the rewarming process. The whole process took 64.7 ± 4.2 min. A paired t-test of the resistivities at the corresponding temperatures for the cooling and rewarming processes showed that *p*>0.10 for the corresponding temperatures, and at a significance level of *α* = 0.05, the resistivity data at the corresponding temperatures for the two processes were not statistically different. The same conclusion can be drawn for the imaginary part of the resistivity at each temperature.

### Temperature dependence of the dielectric properties of blood

These studies confirmed that the dielectric properties of blood were not significantly affected by cooling and rewarming processes. To simplify the experimental procedure, we investigated the variation of the dielectric parameters with frequency during the warming process in eight blood samples, taking 38.2 ± 3.4 min.

As shown in Figures 4C,D, the real and imaginary parts of the blood resistivity changed with frequency at each temperature (the error bars are the standard deviations). It can be observed that although the real and imaginary parts of the resistivity at each temperature were different, the frequency response characteristics were similar. At low frequencies, the real part of the resistivity remained smooth and did not change significantly with frequency; after a few hundred kHz, the real part of resistivity decreased approximately linearly, and after 20 MHz, it decreased at a greater slope. It was also observed that as the temperature increased, the real part decreased at the same frequency point, while the critical frequency point at which the linear decrease occurred gradually increased. The imaginary part was close to zero at low frequencies (10–6.31 kHz), where the standard deviation was relatively large and the data were less consistent and became more consistent at > 6.31 kHz. The imaginary part increased negatively from 6.31 kHz to 1 MHz, then it slowly decreased negatively until approximately 10 MHz, and increased again negatively as the frequency increased. It was also observed that, as the temperature increased, the imaginary part decreased negatively at the same frequency point, and the frequency points of the peak and trough of the imaginary part gradually increased.

The resistivity real part vs. temperature at 10 Hz–100 MHz was analyzed and showed a good linear correlation with a coefficient of determination≥0.990. Because the resistivity imaginary part tended to be close to zero at low frequencies (10 Hz–6.31 kHz), the relative error was large; therefore, we only curved fit the resistivity imaginary part vs. temperature at 6.31 kHz–100 MHz. The results also showed a linear correlation between the imaginary part and temperature at each frequency, with a coefficient of determination≥0.969. We borrowed the method of (Jaspard and Nadi 2002) and used Eq. 4 to calculate the temperature coefficients of the real and imaginary parts of the blood resistivity at each frequency.

$$\mathrm{TC}=\frac{X_{\left(37\right)}-X_{\left(17\right)}}{20*X_{\left(37\right)}}*100\%$$ (4)

where *X*_(37)_ and *X*_(17)_ are the real part (*ρ*_*re*_) and imaginary part (*ρ*_*im*_) of the resistivity at 37°C and 17°C, respectively.

**FIGURE 5:**
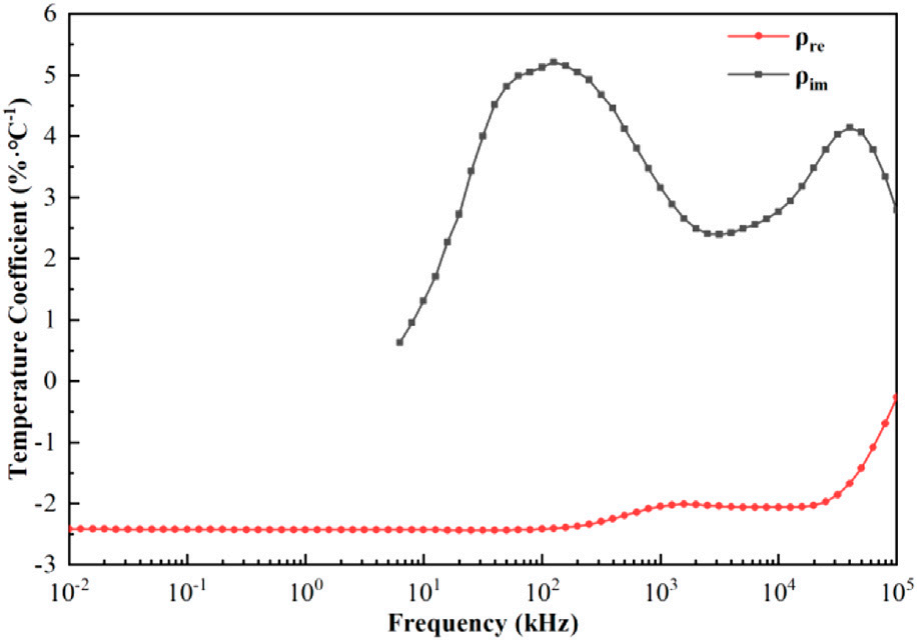
Temperature coefficients of the real and imaginary parts of resistivity as a function of frequency.

Figure 5 showed the variation of the temperature coefficients of the real and imaginary parts of the blood resistivity with frequency; it could be observed that the temperature coefficient of the real part decreases negatively and monotonically, remaining constant from 10 Hz to 100 kHz (−2.42%/°C), decreasing slightly to −2.01%/°C from 100 kHz to 1 MHz, and decreasing with a larger slope to −0.26%/°C after 25 MHz. For the imaginary part of the resistivity, the temperature coefficient was positive and bimodal, with a coefficient of 0.63%/°C at 6.31 kHz, increasing to 5.22%/°C from 6.31 kHz to 126 kHz, decreasing to 2.39%/°C from 126 kHz to 3.16 MHz, and then increasing again to 4.39%/°C from 3.16 MHz to 39.8 MHz. The temperature coefficient finally decreased to 2.79%/°C after 39.8 MHz.

### Temperature-dependent broadband dielectric parameter model

The four equivalent circuits shown in Figure 2 were developed to describe the frequency dependence of the resistivity of blood at 37°C in the range of 10 Hz–100 MHz. The relative error of the resistivity absolute value fitted by the circuit shown in Figure 2A was 5.44%. The values of the components extracted by the circuit shown in Figures 2C,D were negative, which was inconsistent with the actual situation. The relative error of fitting by the circuit shown in Figure 2B was 1.05%, which indicated a good fitting effect. Therefore, we used the equivalent circuit shown in Figure 2B containing three parallel resistors and capacitors, to describe the frequency dependence of the complex resistivity at each temperature.

After determining that the equivalent circuit shown in Figure 2B had the best fit, in order to extract the characteristic parameters related to physiology and pathology, we established a third-order function model more consistent with this study based on the Cole-Cole model (Díaz Pacheco et al., 2021). The model could represent the frequency dependence of the blood complex resistivity at each temperature, as shown in Eq. 5.

$$\rho \left(\mathrm{f,T}\right)=\rho_{\infty }\left(T\right)+\sum_{i=1}^{3}\frac{\rho_{i}\left(T\right)}{1+j\frac{f}{f_{\mathrm{ci}}\left(T\right)}},\;\;f_{ci}=\frac{1}{2*\mathrm{PI*}\rho_{i}\left(T\right)*C_{i}\left(T\right)}$$ (5)

Based on this model, the characteristic parameters related to physiopathology can be extracted, where *ρ*_*∞*_(*T*) is the high-frequency resistivity, *ρ*_*i*_(*T*) is the resistivity change in the *ith* dispersion region (*i* = 1,2,3), *f*_*ci*_(*T*) is the characteristic frequency of the *ith* dispersion region (*i* = 1,2,3), and *ρ*_*0*_(*T*) *= ρ*_*∞*_(*T*) *+ ρ*_*1*_(*T*) *+ ρ*_*2*_(*T*) *+ ρ*_*3*_(*T*) denotes the resistivity under DC, which is related to the substances in the extracellular fluid.

**FIGURE 6:**
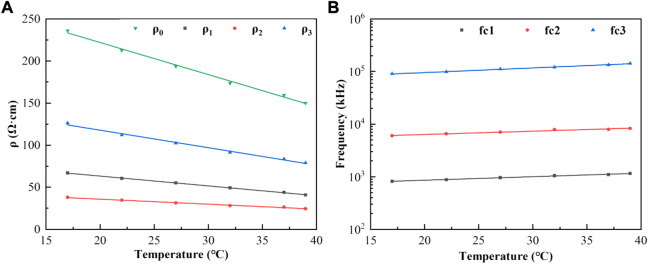
**(A)**: Characteristic parameters ρ_0_, ρ_1_, ρ_2_, ρ_3_ (points) as a function of temperature and linear fit (lines in corresponding colors); **(B)**: Characteristic frequencies f_c1_, f_c2_, f_c3_ (points) as a function of temperature and linear fit. (lines in corresponding colors).

Curve fitting was performed on the relationship between each characteristic parameter and temperature, and the results showed that it had a good linear correlation. The coefficients of determination were all≥0.970, and their linear fitting was shown in Figures 6A,B. The coefficients of the linear fitting equations for each parameter and temperature were listed in Table 1 **.**

**TABLE 1:** Coefficients of linear fit to temperature-dependent function parameters.

Characteristic parameters	n	An	Bn	Coefficient of determination
${\text{ρ}}_{0,fit}$	1	−3.8153	298.29	0.995
${\text{ρ}}_{1,fit}$	2	−1.1658	86.524	0.999
${\text{f}}_{c1,fit}$	3	15006	556709	0.995
${\text{ρ}}_{2,fit}$	4	−0.5959	47.704	0.991
${\text{f}}_{c2,fit}$	5	−3.8505	300.5	0.970
${\text{ρ}}_{3,fit}$	6	−2.0835	159.5	0.992
${\text{f}}_{c3,fit}$	7	2×10^6^	5×10^7^	0.986

Finally, the relationship between the complex resistivity, frequency, and temperature could be established as Eq. 6:

$$\rho \left(\mathrm{f,T}\right)=0.0299*T+\frac{-1.1658*T+86.524}{1+j\frac{f}{15006*T+556709}}+\frac{-0.5959*T+47.704}{1+j\frac{f}{-3.8505*T+300.5}}+\frac{-2.0835*T+159.5}{1+j\frac{f}{2*10^{6}*T+5*10^{7}}}+4.562$$ (6)

(*T* is any temperature from 17°C to 39°C, *f* is any frequency from 10 Hz to 100 MHz, and the unit of complex resistivity *ρ* is Ω·cm).

**FIGURE 7:**
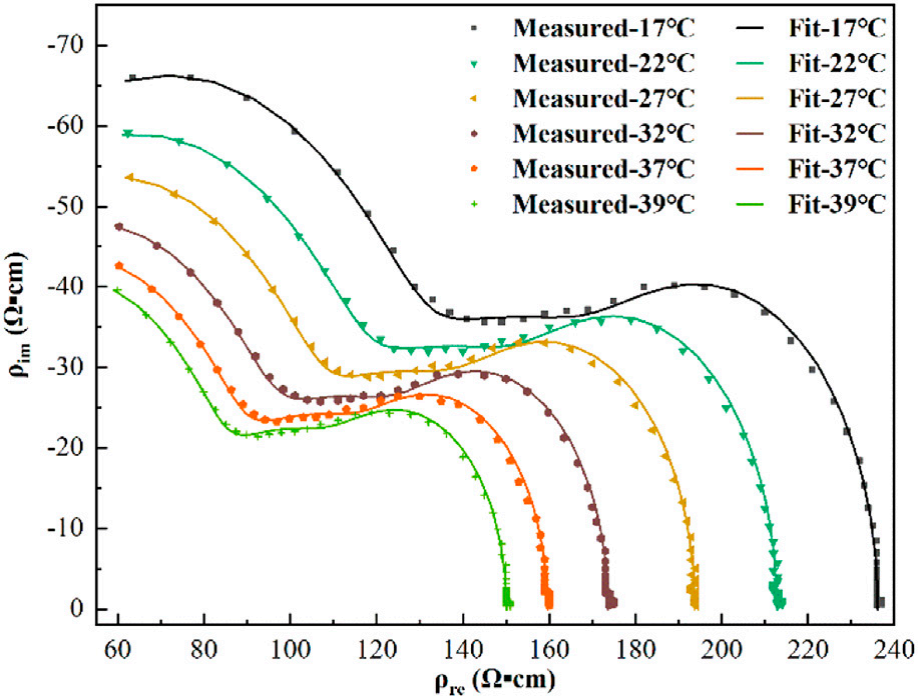
Resistivity measurements (points) and third-order function model fit (lines) at each temperature.

As shown in Figure 7, the points in the figure were the measured resistivity values at each temperature, and the solid lines were the simulated values calculated using Eq. 6. The maximum relative error between the simulated values calculated according to Eq. 3 and the measured values was 1.39%, and there was good agreement between the measured and fitted values.

## Discussion

The measurement accuracy is decisive in the study of the dielectric properties of active tissues. The temperature dependence of the blood dielectric properties at 10 Hz–100 MHz were poorly studied owing to electrode polarization effects in the low-frequency range (below tens of kHz) and the distribution parameters of the inter-conductor in the high-frequency range (above several hundred kHz). Therefore, we proposed using the four/two-electrode method to integrate the data to achieve an accurate measurement of the broadband dielectric parameters. The four-electrode method was used to overcome the influence of electrode polarization at low frequencies, and the two-electrode method was used at high frequencies to reduce the influence of inter-electrode distribution parameters. This system was used to measure the conductivities of saline at different temperatures between 10 Hz and 100 MHz. The maximum relative error between the results and theoretical values (Peyman et al., 2007) was only 1.73%, indicating the high accuracy of the developed system and the credibility of the results. In addition, the dielectric properties of blood at 37°C were compared with (Wolf et al., 2011) and (Gabriel et al., 1996). The resistivity values below 1 MHz measured by Wolf do not truly reflect the dielectric properties of the blood because of the presence of electrode polarization (Takahashi et al., 2022) in the low-frequency range.

Active tissues are progressively denatured and become necrotic after isolation. Whether the dielectric parameters of anticoagulant blood are affected by the time of isolation, resulting in temperature-controlled experiments that do not accurately reflect the temperature dependence of the blood dielectric parameters, has rarely been investigated in previous studies. Therefore, we also investigated the time dependence of the dielectric properties of isolated blood. It could be found that: the maximum relative rate of change at 100, 140, 160, and 220 min of isolation are 1.85%, 2.87%, 3.31%, and 5.14% respectively. The overall amount of change was relatively small, especially within 100 min of isolation, where the rate of change did not exceed 1.85%. To minimize the effect of separation time on the dielectric properties, this study used a specially designed impedance measurement box with high heat transfer efficiency combined with a temperature-controlled water sink to achieve rapid temperature control of the samples, thus enabling us to control the temperature-dependent experiments on the dielectric properties of blood within 100 min.

In addition to local cryotherapy, existing treatments, such as hypothermia (Ho et al., 2021) or vital organ protection, such as aortic arch replacement surgery (Salem et al., 2020) in which the patient’s body temperature is lowered to a minimum of around 17°C (Pastukhov et al., 2016), rarely reduces the patient’s body temperature below 17°C; additionally, considering that excessive temperatures may lead to the occurrence of blood cell rupture and hemolysis. Therefore, in this study, the dielectric properties of blood samples were measured and analyzed at six temperatures:17, 22, 27, 32, 37, and 39°C. As it was uncertain whether the cooling and rewarming processes would have a significant irreversible effect on the dielectric properties of blood, this study also measured and compared the differences in the dielectric properties of blood at each temperature between cooling and rewarming. The results showed no significant difference in the dielectric properties of blood at the same temperature in either process, indicating that the effect of the process on the physiological state of blood is reversible. This finding suggested that the experimental procedure could be further simplified by only studying the blood dielectric parameters during unidirectional temperature changes to minimize the total measurement time and ensure the stability of the blood dielectric parameters.

The frequency response characteristics of the real and imaginary parts of blood resistivity at each temperature were similar, with the real part remaining unchanged at low frequencies. The real part decreased in an approximately linear manner after a few hundred kHz, and decreased at a greater slope after approximately 20 MHz, with two obvious dispersion phenomena. The imaginary resistivity value was close to zero at low frequencies (10–6.31 kHz) because the imaginary value was too small to be affected by measurement errors, and the consistency of the imaginary data in this frequency range was poor. As the frequency increased, the imaginary part showed a peak at approximately 1 MHz and a valley near 10 MHz, with the blood complex resistivity demonstrating a clear frequency dependence. In addition, the temperature had a significant effect on the blood dielectric parameters; as the temperature increased, the real part of the resistivity decreased, and the frequency turning point at which the real part decreased linearly increased.

In order to more intuitively study the change law of blood dielectric parameters affected by temperature and eliminate the interference of other influencing factors, we extracted the temperature coefficient based on the conclusion that the dielectric parameters of blood were linearly related to temperature. From Figure 5, the temperature coefficients of the dielectric properties of blood as a function of frequency showed that the temperature coefficient of the real part of the resistivity at low frequencies (10–100 kHz) remains constant (−2.42%/°C). This might be because the blood plasma had a strong ionic conductivity due to the abundance of chloride and sodium plasma, and when low-frequency currents passed through the blood, they mainly flowed through the plasma; therefore, the blood showed good pure resistive properties in this frequency range (Castro-Giráldez et al., 2010). The temperature in this frequency range mainly affected the degree of ionization in the plasma, which was not significantly frequency-dependent; therefore, the temperature coefficient of the real part of the resistivity remained constant in this frequency range. As the frequency increased, the current began to partially crossed the cell membrane, at which point Maxwell-Wagner type dispersions were generated at the plasma-cell membrane and cell membrane-cytoplasm interfaces, which had different dielectric properties (Foster and Schwan, 2019). Because the temperature in this frequency band affected the dielectric properties of the plasma, cell membrane, and cytoplasm, the absolute value of the temperature coefficient of the real part began to decrease to -2.01%/°C at 1 MHz. The temperature coefficient of the imaginary part showed an increase and then decreases with increasing frequency from 6.3 kHz to 3.16 MHz, with a maximum value of 5.22%/°C at 126 kHz, and then decreased to 2.39%/°C at 3.16 MHz. As the frequency continued to increase, the current through the cell membrane exhibited high conductivity characteristics, and the current interacted with hemoglobin in the red blood cell to produce δ dispersion (Zhbanov and Yang, 2020). At this point, the temperature coefficients of the real and imaginary parts of the resistivity were significantly reduced, and they were −0.26%/°C and 2.79%/°C, respectively, at 100 MHz.

To further quantify the relationship between the blood dielectric parameters, frequency, and temperature, extract the characteristic parameters closely related to physiology and pathology and study their changes with temperature, we comprehensively compared and analyzed the fitting effects of the four equivalent circuits shown in Figure 2. The relative error in fitting the equivalent circuit, shown in Figure 2B is 1.05%, which was a better fit than the other equivalent circuits and better matched the actual characteristic parameters; the equivalent circuit containing three parallel resistors and capacitors was determined to describe the frequency dependence of the dielectric properties of blood and was used to develop a third-order function model. The two Maxwell-Wagner type dielectric relaxations (Andoulsi-Fezei et al., 2018) at the plasma-cell membrane and cytoplasm-cell membrane interfaces could be represented by the parallel resistances ρ_1_ and ρ_2_–capacitances C_1_ and C_2_, respectively, and the δ dispersion could also be represented by the parallel resistance ρ_3_–capacitance C_3_. Based on this model, we further analyzed the variation in the model parameters with temperature, and the results were shown in Figures 6A,B. It could be observed that each parameter varied linearly with temperature: *ρ*_*i*_ (*i* = 1, 2, 3) decreased linearly with increasing temperature, while *f*_*i*_ (*i* = 1, 2, 3) increased linearly. The temperature dependence of *ρ*_*i*_ was related to the temperature dependence of the ionic components of the plasma and blood cells: the higher the temperature, the stronger the ionization, and the lower the resistivity of the corresponding components. *f*_*i*_ might be related to the combined effect of the membrane capacitance and electrolyte in each sector. After establishing a linear relationship between each characteristic parameter and temperature, a third-order function model was obtained to characterize the relationship between resistivity, temperature, and frequency. The fitting results showed that the maximum error between the model data and measured data was less than 1.39%, which can better reflect the variation in the dielectric properties with frequency and temperature.

This study accurately established a third-order function model between the blood complex resistivity, temperature, and frequency, which had the following benefits:1. The concise formula covered seven orders of magnitude of data and accurately established the relationship between the three, allowing researchers to calculate the blood dielectric parameters at any temperature and frequency in the range of 17–39°C and 10–100 MHz; 2. The biophysical laws of the temperature affecting the dielectric properties of blood have been understood, providing more information about the parameters related to tissue physiology and pathology, and providing new ideas for studying the temperature dependence of other biological tissue dielectric parameters; 3. Facilitated subsequent electron simulation and provides basic data for the integration and modeling of the relationship between dielectric parameters, temperature, and frequency in various tissues and organs; 4. This provided the basis for the calculation of the specific absorption rate, cryotherapy, biosensor design, noninvasive temperature monitoring, and other technologies.

## Conclusion

To achieve accurate measurement of the broadband dielectric parameters of blood and to overcome the effects of low-frequency electrode polarization and high-frequency stray capacitance, this study adopted a composite impedance measurement box that can realize four/two-electrode measurement and integrated the data from the four/two-electrode measurement to ensure the accuracy of the dielectric property measurement. In this study, based on the study of the time dependence of the dielectric properties of blood after isolation, the effect of isolation time on the temperature dependence of blood dielectric parameters was minimized by limiting the total measurement time to 100 min. A comparative analysis of the blood dielectric properties during cooling and rewarming confirmed that there was no significant difference in the temperature dependence of the blood dielectric properties between the two processes. Based on this, the variation regularity of the blood dielectric properties at a temperature of 17–39°C was measured and analyzed, and the temperature coefficients of the dielectric parameters were extracted. Finally, the study established a third-order function model (Eq. 6) that reflected the relationship between blood resistivity, frequency, and temperature. The fitting error of this model was less than 1.39%, which could provide basic theoretical and data support for studying the biophysical effects of temperature. This study can be used as a basis for calculating the specific absorption rate, noninvasive temperature monitoring, biosensor design, monitoring, and early warning of brain injury under variable temperatures in cardiac surgery in our group.

There are some limitations in this study. Firstly, the low frequency (10 Hz–6.3 kHz) measurements of the blood resistivity are significantly affected by measurement errors, resulting in poor data consistency. Secondly, there is controversy as to whether anticoagulants have a significant effect on the dielectric parameters of blood (Wolf et al., 2011; Salahuddin et al., 2017a; Salahuddin et al., 2017b; Dunne et al., 2019). Therefore, we will continue to investigate how to minimize measurement errors at low frequencies and the effect of anticoagulants on the blood dielectric parameters. Specifically, we will investigate the dielectric properties of pure blood, the influence of anticoagulant type and dose on blood dielectric parameters, and the change rule of dielectric parameters of anticoagulant blood with time in the following research.

## Data Availability

The original contributions presented in the study are included in the article/Supplementary Material, further inquiries can be directed to the corresponding authors.
