# Supplementary figures and images for: Temperature dependence of dielectric properties of blood at 10 Hz–100 MHz
Source: Front Physiol. 2022 Oct 26;13:1053233. doi: 10.3389/fphys.2022.1053233 (PMC9644111; doi:10.3389/fphys.2022.1053233)

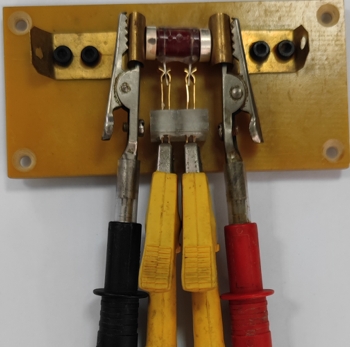

Supplement: Supplementary file 1 [file Image3.JPEG]

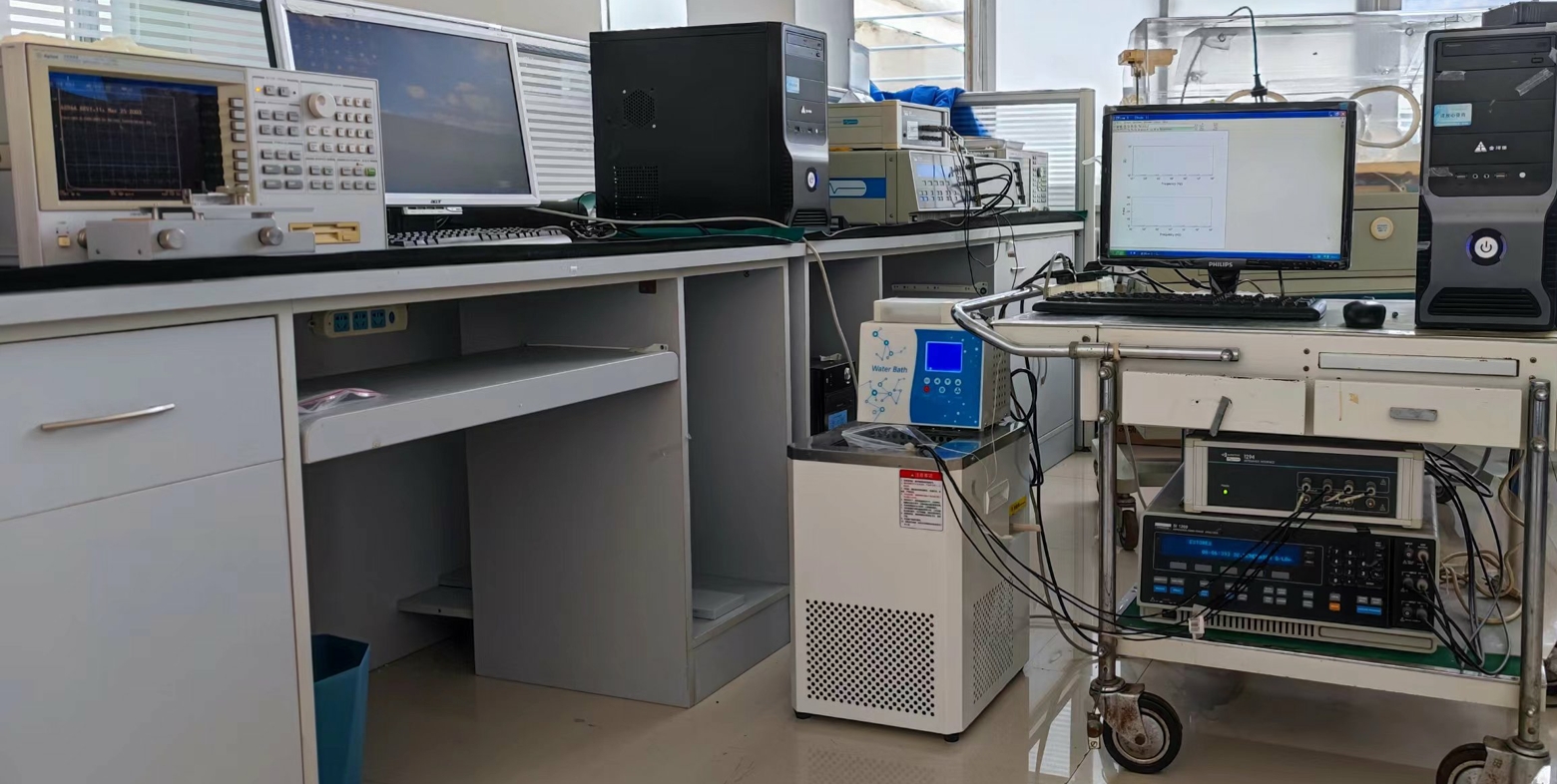

Supplement: Supplementary file 5 [file Image4.JPEG]

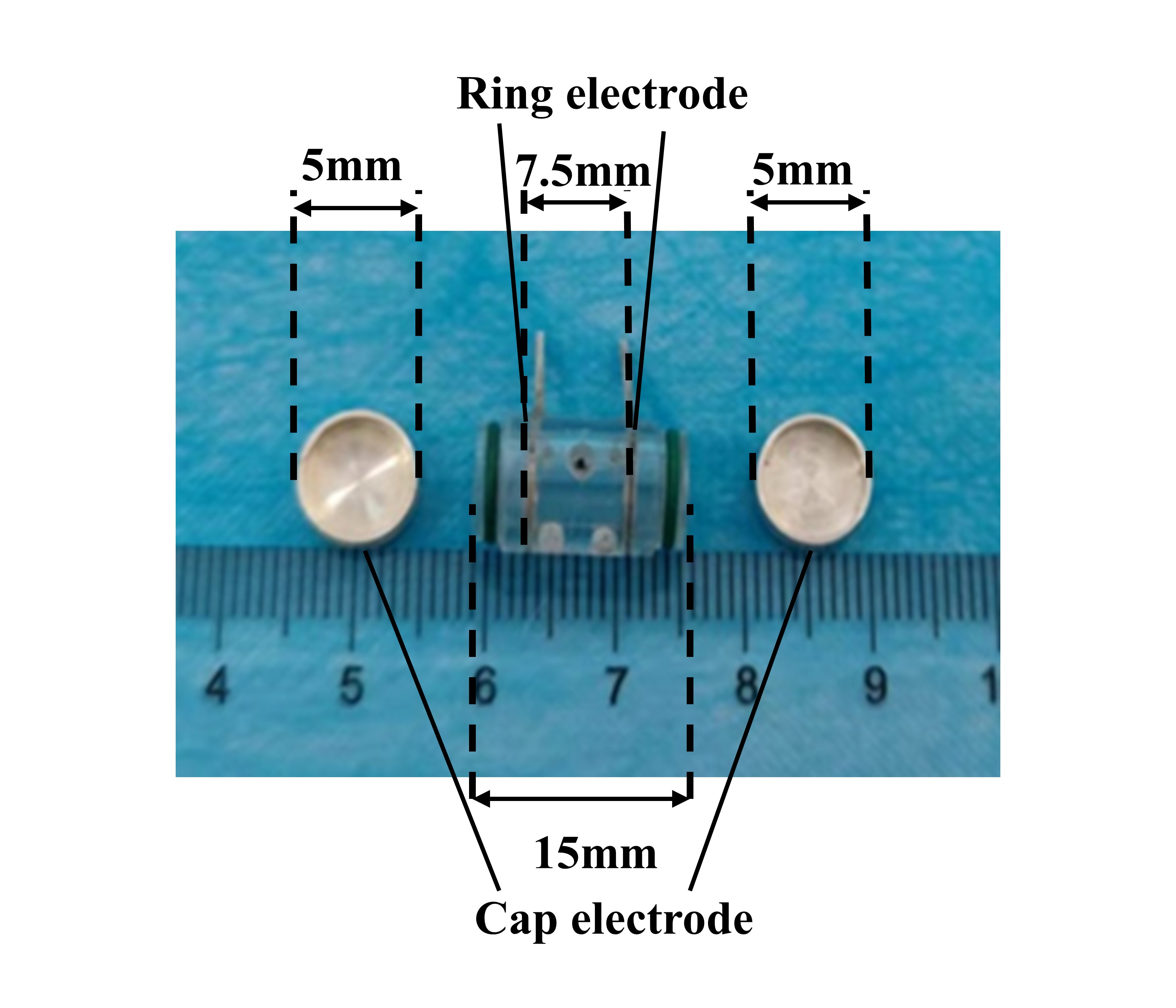

Supplement: Supplementary file 7 [file Image2.JPEG]

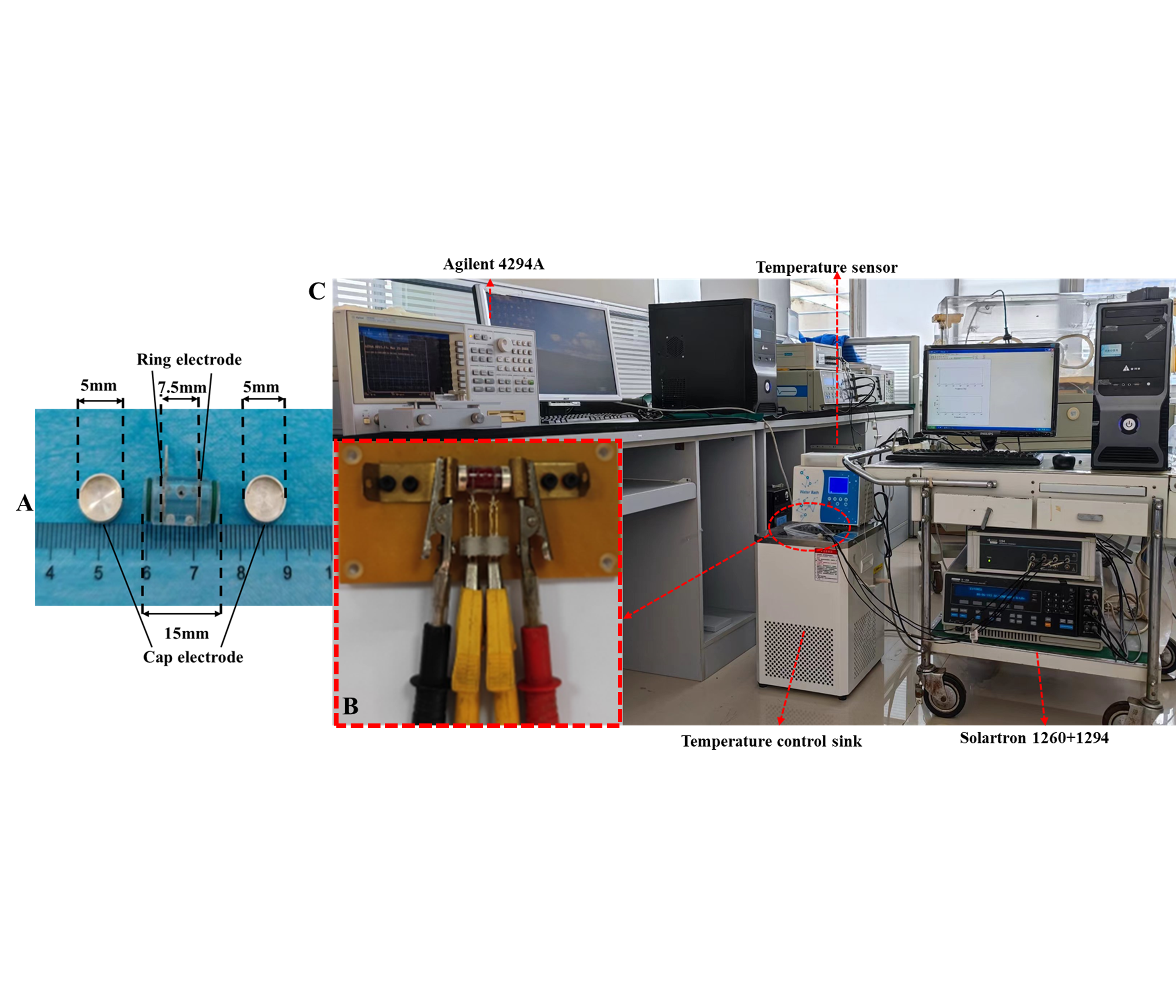

Supplement: Supplementary file 9 [file Image1.TIF]

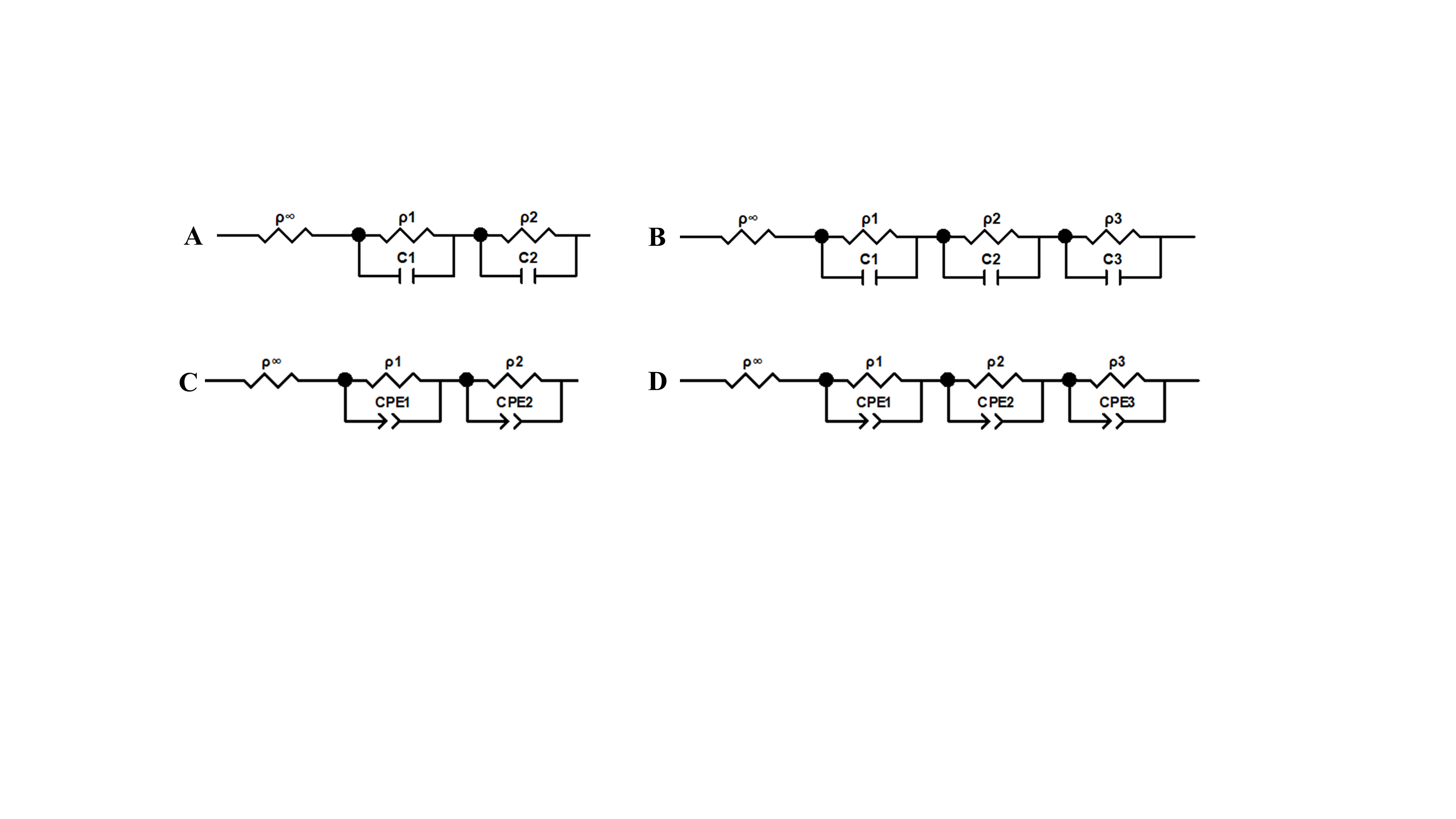

Supplement: Supplementary file 13 [file Image5.TIF]
