# Supplementary material for: Temperature dependence of dielectric properties of blood at 10 Hz–100 MHz
Source: Front Physiol. 2022 Oct 26;13:1053233. doi: 10.3389/fphys.2022.1053233 (PMC9644111; doi:10.3389/fphys.2022.1053233)
Supplement: Supplementary file 16 [file DataSheet1.docx]

Supplementary Material

# Supplementary Figures and Tables

## Supplementary Figures





**Supplementary Figure 1. Changes of physiological saline conductivity with frequency at various temperatures.**





**Supplementary Figure 2. Change of the real part of blood resistivity with time at various frequencies.**

## Supplementary Tables

Supplementary Table S1. Conductivities of physiological saline at various temperatures.

| Temperatures /°C | Conductivities- theory / (S·m^-1^ ) | Conductivities- experiment / (S·m^-1^ ) | Relative error (%) |
| --- | --- | --- | --- |
| 17 | 1.33 | 1.34 | 0.75 |
| 22 | 1.46 | 1.47 | 0.68 |
| 27 | 1.60 | 1.62 | 1.25 |
| 32 | 1.73 | 1.76 | 1.73 |
